# Phys-Mamba: Physics-informed selective state-space fusion network for high-fidelity underwater image restoration

**DOI:** 10.1371/journal.pone.0354030

**Published:** 2026-09-02

**Authors:** Hongwei Li, Weishen Li, Yuqing Lao, Jie Li, Junjie He, Caihong Wu, Huilong Zhong, Shaoji Huang

**Affiliations:** 1 Guangdong Communication Polytechnic, Guangzhou, China; 2 Hubei University of Arts and Science, Xiangyang, China; Zhejiang University, CHINA

## Abstract

Underwater image restoration is fundamental for enhancing the visual perception of Autonomous Underwater Vehicles (AUVs) and Remotely Operated Vehicles (ROVs). However, existing methods face a persistent trade-off: GAN-based approaches (e.g., FUnIE-GAN) can introduce physically inconsistent artifacts due to the absence of explicit optical constraints; Transformer architectures achieve global modeling but suffer from quadratic computational complexity *O*(*N*^2^), limiting their suitability for real-time multi-scale and 4K underwater imagery; and several recent Mamba-based methods still use physical or degradation priors mainly as auxiliary guidance. In this paper, we propose Phys-Mamba, a physics-informed selective state-space fusion network for high-fidelity multi-scale underwater image restoration. Our method introduces two core innovations: (1) we encode UIFM-inspired latent degradation variables and use them to modulate Mamba’s selective scan mechanism (Δ, *B*, *C*) via a Physics Embedding Module, encouraging consistency with the UIFM formulation and helping suppress color distortion and over-enhancement; (2) we design a Dynamic Cross-Scale Fusion Module (CSFM) that adaptively aggregates multi-scale features without channel explosion, enabling detail recovery from low-resolution semantics to 4K textures while maintaining linear *O*(*N*) complexity. Extensive experiments on EUVP, UCHN, Synthetic Deep-Sea, and LSUI benchmarks show that Phys-Mamba achieves competitive restoration performance, with PSNR of 28.12 dB and SSIM of 0.915 on EUVP, while maintaining a compact model size of 8.6M parameters. On an RTX 4090 with FP16 precision and batch size 1, the model reaches 34 FPS for 4K inference. When used as a preprocessing module, it improves YOLOv8 mAP@0.5 by 20.7 percentage points in our evaluation setting. These results suggest that UIFM-inspired selective-scan modulation is a promising direction for balancing restoration quality, global modeling, and high-resolution inference efficiency in underwater robotic vision.

## Introduction

With the increasing strategic significance of ocean exploration and underwater resource exploitation, the visual perception capabilities of Autonomous Underwater Vehicles (AUVs) and Remotely Operated Vehicles (ROVs) have become a core focus in both academia and industry [1–5]. Limited by the complex underwater optical environment, light suffers from severe frequency-selective absorption and forward/backward scattering during propagation [6–11]. These physical degradation effects directly lead to severe color distortion (predominantly blue-green casts), loss of contrast, detail blurring, and reduced visibility in captured imagery [3,5]. Consequently, recovering physically consistent high-fidelity visual information from degraded observations is not only a prerequisite for image processing pipelines but also a critical foundation for robust downstream vision tasks, including object detection, semantic segmentation, and simultaneous localization and mapping (SLAM) [1–5].

Early underwater image restoration primarily relied on classical physical imaging models or hand-crafted priors, treating restoration as an inverse problem to jointly estimate the transmission map and ambient light [6–12]. Representative approaches encompass variants of the Dark Channel Prior (DCP) [12], wavelength compensation [9], color attenuation priors [8], initial single-image dehazing techniques [10], and weighted variational models for simultaneous reflectance and illumination estimation [11]. However, single-prior assumptions exhibit poor generalization across heterogeneous water bodies, while the ill-posed nature of parameter estimation and prohibitive computational cost severely limit real-time applicability on resource-constrained underwater platforms [5,6,10].

To overcome generalization limitations, data-driven CNN- and GAN-based methods demonstrated powerful nonlinear mapping capabilities from paired or unpaired data [1–4,13–15]. Prominent examples include FUnIE-GAN [1], WaterGAN [4], multi-scale fusion networks [15], and medium transmission-guided multi-color space embedding approaches [3]. Although these models significantly improved processing speed and visual quality, their lack of explicit physical constraints frequently generates physically inconsistent artifacts and false structural distortions in unknown or turbid waters [3,5], restricting industrial reliability.

In recent years, Transformer architectures advanced visual restoration through global dependency modeling [16–20]. The U-shape Transformer [16] integrates channel- and spatial-wise attention to emphasize severely attenuated regions, while efficient variants such as Swin Transformer [19] and Restormer [20] were explored to mitigate memory overhead. Nevertheless, the inherent quadratic complexity *O*(*N*^2^) of self-attention [17] increases memory consumption and latency for multi-scale and especially 4K underwater imagery, motivating more efficient sequence modeling strategies [5,16].

To address the trade-off among physical fidelity, global modeling capability, and computational efficiency, this paper proposes Phys-Mamba, a physics-prior-guided selective state-space fusion network. Unlike prior Transformer models, Phys-Mamba employs the selective state-space model (Mamba) [21] with linear complexity *O*(*N*) as the fundamental operator. To our knowledge, it is one of the first attempts to explicitly modulate Mamba selective-scan parameters using UIFM-inspired priors by transforming latent degradation variables into a Physics Embedding that modulates Δ, *B*, and *C*. This design encourages the sequence modeling process to remain consistent with the simplified UIFM formulation [6,7]. Additionally, the proposed Dynamic Cross-Scale Fusion Module (CSFM) adaptively aggregates multi-scale features without channel explosion, enabling detail recovery from low-resolution semantics to 4K textures while preserving linear complexity.

Experiments on EUVP [5], UCHN, Synthetic Deep-Sea, and LSUI benchmarks show that Phys-Mamba achieves competitive restoration performance with 8.6M parameters and 34 FPS 4K inference on an RTX 4090, while improving downstream YOLOv8 mAP@0.5 by 20.7 percentage points in the evaluated preprocessing setting. These results suggest that Phys-Mamba partially alleviates the trade-off among physical fidelity, global modeling, and high-resolution inference efficiency for underwater robotic vision.

The main contributions of this paper are summarized as follows:

- **Explicit parameter-level coupling between UIFM-inspired priors and Mamba selective scan:** We propose a physics-informed Mamba restoration framework in which UIFM-inspired latent degradation variables are transformed into embeddings that modulate the selective-scan parameters Δ, *B*, and *C*. This design differs from prior degradation-aware Mamba methods by introducing the prior at the state-space parameter level rather than only as auxiliary feature guidance or post-processing.
- **Physics-guided selective scan with dynamic cross-scale fusion:** We introduce a Physics-Guided Selective Scan mechanism and a Dynamic Cross-Scale Fusion Module (CSFM) to combine global sequence modeling with multi-scale feature aggregation while preserving linear computational complexity.
- **Compact high-resolution inference and downstream evaluation:** The proposed model achieves competitive restoration results with 8.6M parameters and 34 FPS 4K inference on an RTX 4090. We further evaluate its effect as a preprocessing module for underwater object detection.

## Related work

**Fig 1:**
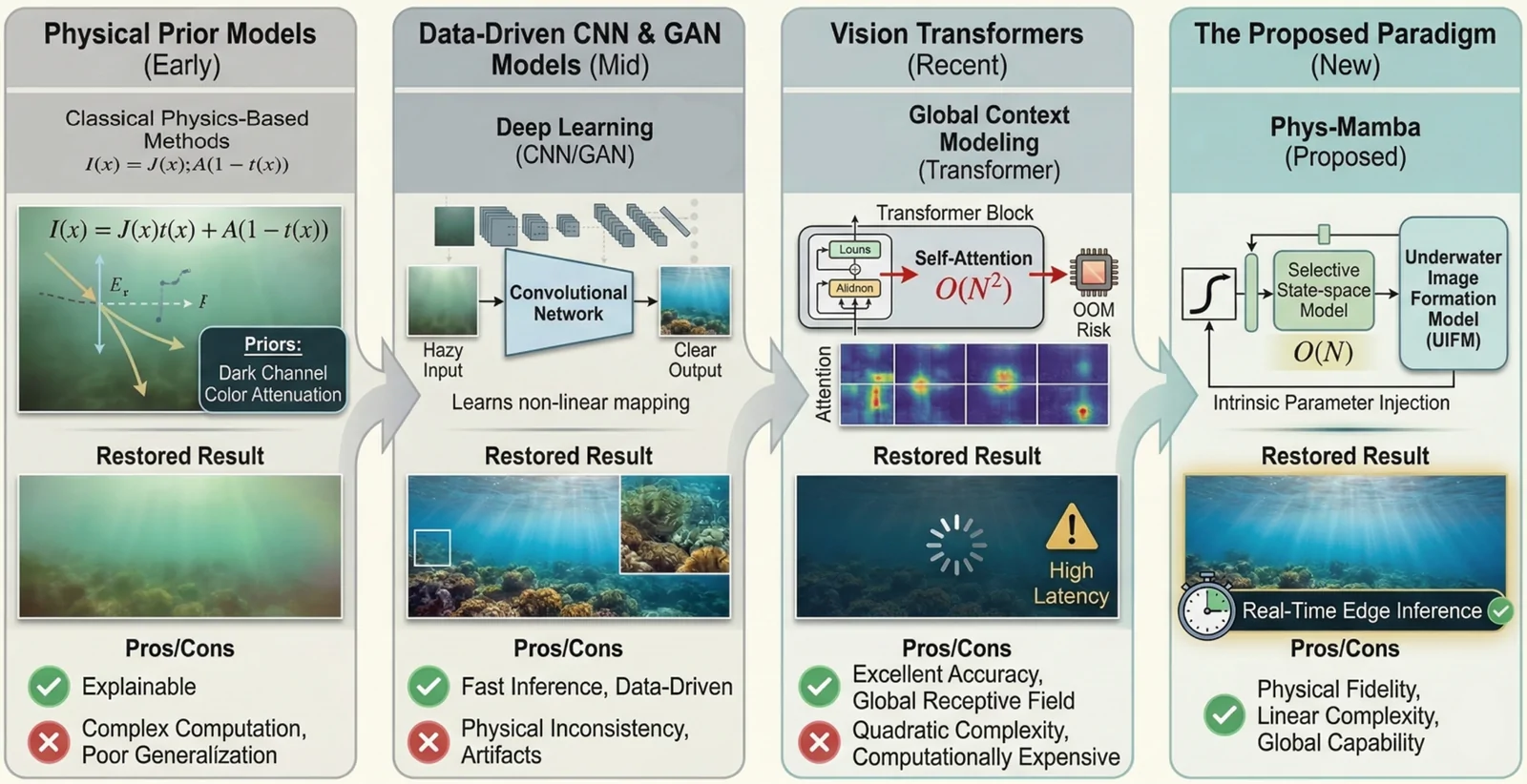
Evolution comparison of research paradigms in underwater image restoration.

Underwater Image Restoration (UIE) aims to overcome frequency-selective absorption and severe scattering to recover high-fidelity visual information for AUVs and ROVs. As illustrated in Fig 1, the research paradigm has evolved from physical prior models and data-driven CNN/GAN approaches to globally modeled Transformer architectures and, most recently, efficient state-space models. However, existing methods continue to face a trade-off among physical fidelity, global modeling capability, and computational efficiency. The Phys-Mamba proposed in this paper is designed to partially alleviate this trade-off (see the evolution comparison in Fig 1).

### Physical models and visual priors

Early underwater image restoration mainly relied on classical physical imaging models or hand-crafted priors [6–12]. These methods formulate restoration as an inverse problem to estimate the spatially varying transmission map and global ambient light [6,7]. Representative works include variants of the Dark Channel Prior (DCP) [12], wavelength compensation [9], color attenuation priors [8], initial single-image dehazing techniques [10], and weighted variational models for simultaneous reflectance and illumination estimation [11]. As noted by Akkaynak and Treibitz [6], single-prior assumptions exhibit poor generalization across heterogeneous water bodies; the ill-posed nature of joint parameter estimation, combined with high computational cost, limits real-time applicability on underwater robots [5,10].

### Data-driven CNN and GAN models

To address generalization limitations, CNN- and GAN-based methods have shown strong potential [1–4,13–15]. These approaches learn nonlinear mappings from paired or unpaired data [13,14]. Notable examples include FUnIE-GAN [1], WaterGAN [4], multi-scale fusion networks [15], and medium transmission-guided multi-color space embedding [3]. While processing speed improved significantly and the benchmark dataset introduced in [5] established standardized quantitative evaluation, the lack of explicit physical constraints leads to artifacts in turbid conditions [3], and local receptive fields hinder capture of long-range dependencies inherent in underwater attenuation [5].

### Vision transformers and state-space models

To overcome CNN locality, self-attention and Transformer architectures were introduced into UIE [16–20]. The U-shape Transformer [16] integrates channel- and spatial-wise attention to focus on severely attenuated regions. Although global context aggregation improves structural accuracy, the *O*(*N*^2^) complexity of self-attention [17] increases memory overhead and latency for 4K images, making deployment-oriented high-resolution inference difficult. Efficient Transformer variants such as Swin Transformer [19] and Restormer [20] have been explored to mitigate this issue, yet the quadratic complexity remains a practical challenge for real-time high-resolution underwater applications [5,16].

Recently, Selective State-Space Models (Mamba) [21] have improved the efficiency of long-range modeling with linear *O*(*N*) complexity. Several Mamba-based UIE methods have subsequently emerged, including UWMamba [22], PixMamba [23], degradation information-guided Mamba (UIEMamba) [24], Mamba-enhanced spectral-attentive wavelet network [25], FACMamba [26], and Mamba-convolution hybrid networks [27]. These works support the efficiency of state-space models and show that degradation-aware, spectrum-aware, or physics-inspired cues can be useful for underwater enhancement.

Several recent Mamba-based underwater enhancement methods have already explored degradation-aware, spectrum-aware, or physics-inspired designs. Our intention is therefore not to claim that physical or degradation priors have never been considered in Mamba-based UIE. The distinction of Phys-Mamba lies in where and how such priors are used. Existing methods mainly introduce degradation information through auxiliary branches, attention or guidance modules, spectral/wavelet feature processing, or loss-level constraints. In contrast, Phys-Mamba uses UIFM-inspired latent estimates to modulate the selective-scan parameters Δ, *B*, and *C* inside the state-space evolution. The proposed contribution should therefore be understood as an explicit parameter-level coupling between an underwater image formation prior and the Mamba selective-scan mechanism.

## Proposed method

### Overall Architecture

**Fig 2:**
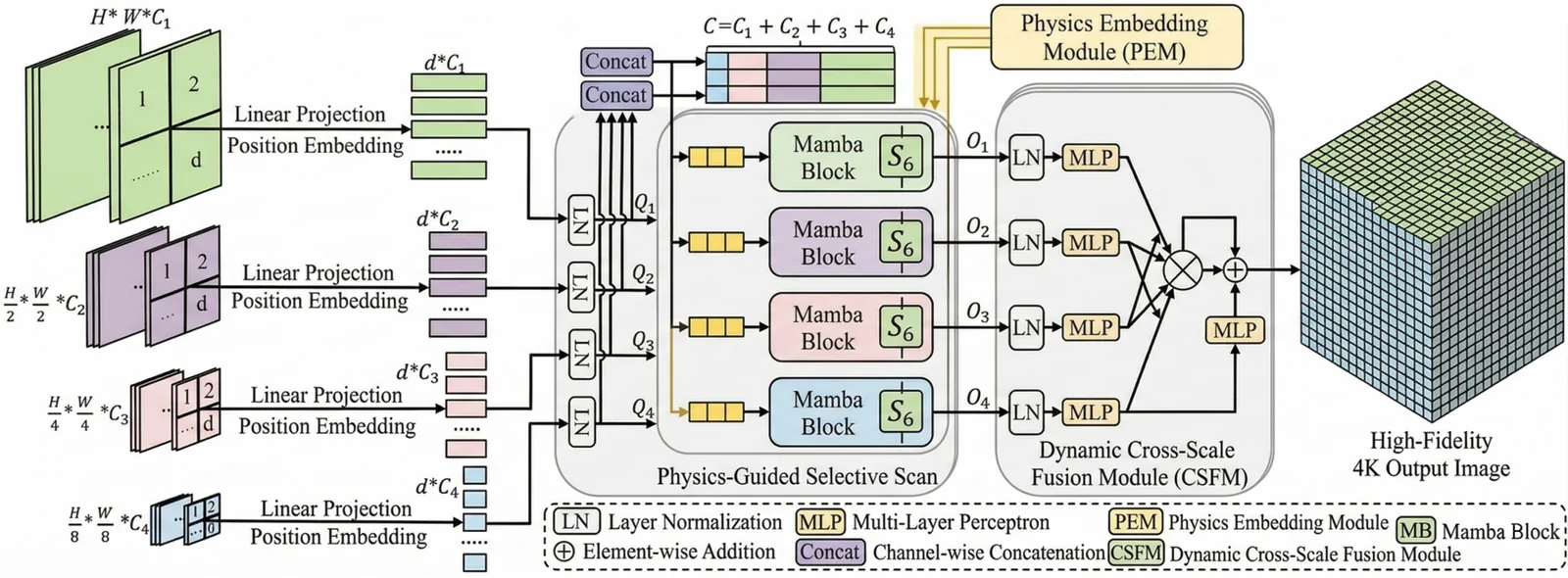
The overall architecture of the proposed Phys-Mamba network.

To reduce the computational overhead of Transformer-based architectures when processing high-resolution underwater images while preserving visual quality and physical consistency, we propose Phys-Mamba, a hierarchical encoder-decoder network as illustrated in Fig 2.

The encoder comprises 4 downsampling stages. Each stage consists of one 3×3 convolution layer (stride 2) followed by 3 stacked Mamba Blocks (hidden dimension 256, state dimension 64, expansion factor 2). In parallel, a lightweight degradation estimation branch, described in the following subsection, extracts UIFM-inspired latent degradation variables from the input. The resulting multi-scale feature sequences, together with the Physics Embedding, are fed into the Physics-Guided Mamba Blocks to perform efficient long-range dependency modeling with linear complexity *O*(*N*). In the decoder phase, we discard conventional skip connections and instead employ the proposed Dynamic Cross-Scale Fusion Module (CSFM) for adaptive multi-scale feature aggregation. The final decoder stage reconstructs a restored image with improved physical consistency.

The entire architecture avoids self-attention, keeps the total parameter count at 8.6M, and enables efficient 4K inference on a desktop-class RTX 4090 GPU. Embedded deployment on AUV/ROV hardware is left for future validation.

### Physics-informed guidance module

Purely data-driven models often generate physically inconsistent artifacts in heterogeneous underwater environments. To regularize the solution space, we adopt the classical Underwater Image Formation Model (UIFM) [6,7], which is applicable to medium-to-low turbidity natural underwater scenes under the linear scattering assumption (nonlinear effects in extreme low-light conditions are not modeled in this study). The model is expressed as:


$$\begin{array}{c} I(x)=J(x)t(x)+A(1-t(x)) \end{array}$$ (1)

where *I*(*x*) is the observed degraded image, *J*(*x*) is the desired clear image, *t*(*x*) is the spatially varying transmission map, and *A* is the global ambient light.

A lightweight 3-layer CNN (3×3 kernels, ReLU activation, average pooling) estimates *t*(*x*) and *A* from downsampled 256×256 inputs. This branch is trained jointly end-*t*o-end with the main network using the same high-fidelity loss. It should be noted that the estimated transmission map and ambient light are not treated as calibrated physical measurements. Since ground-truth transmission and ambient-light annotations are not available in the adopted datasets, they are used as UIFM-inspired latent degradation variables. Their role is to provide scene-adaptive degradation cues for selective-scan modulation and physical reconstruction consistency, rather than to recover exact optical parameters. This clarification prevents overinterpreting the outputs of the lightweight CNN as directly measurable physical quantities. The estimated latent degradation variables are then projected into a high-dimensional continuous Physics Embedding $E_{phys}$ (dimension 256) via a two-layer MLP. This embedding subsequently modulates the core parameters of the selective scan mechanism, encouraging the sequence modeling process to remain consistent with the simplified UIFM formulation.

To further enforce physical consistency during training, we introduce an explicit physical reconstruction loss:


$$\begin{array}{c} {ℒ}_{phys}=\|I-(\hat{J}⊙\hat{t}+\hat{A}⊙(1-\hat{t})){\|}_{1}, \end{array}$$ (2)

In Eq. (2), *I* denotes the observed degraded image, $\hat{J}$ is the restored image predicted by the main restoration network, $\hat{t}$ is the estimated transmission map, and $\hat{A}$ is the estimated ambient-light component. The operator ⊙ denotes element-wise multiplication, and $\|\cdot {\|}_{1}$ denotes the mean absolute reconstruction error. The term $\hat{J}⊙\hat{t}+\hat{A}⊙(1-\hat{t})$ reconstructs the degraded observation according to the forward UIFM process. Minimizing ${ℒ}_{phys}$ therefore encourages the restored image and the estimated degradation variables to remain mutually consistent under the assumed image-formation model. This loss does not guarantee exact recovery of physical parameters, but it reduces the degree of freedom of the restoration network and discourages arbitrary color or contrast transformations that cannot reproduce the input observation through the UIFM approximation.

### Physics-guided selective state-space model

**Fig 3:**
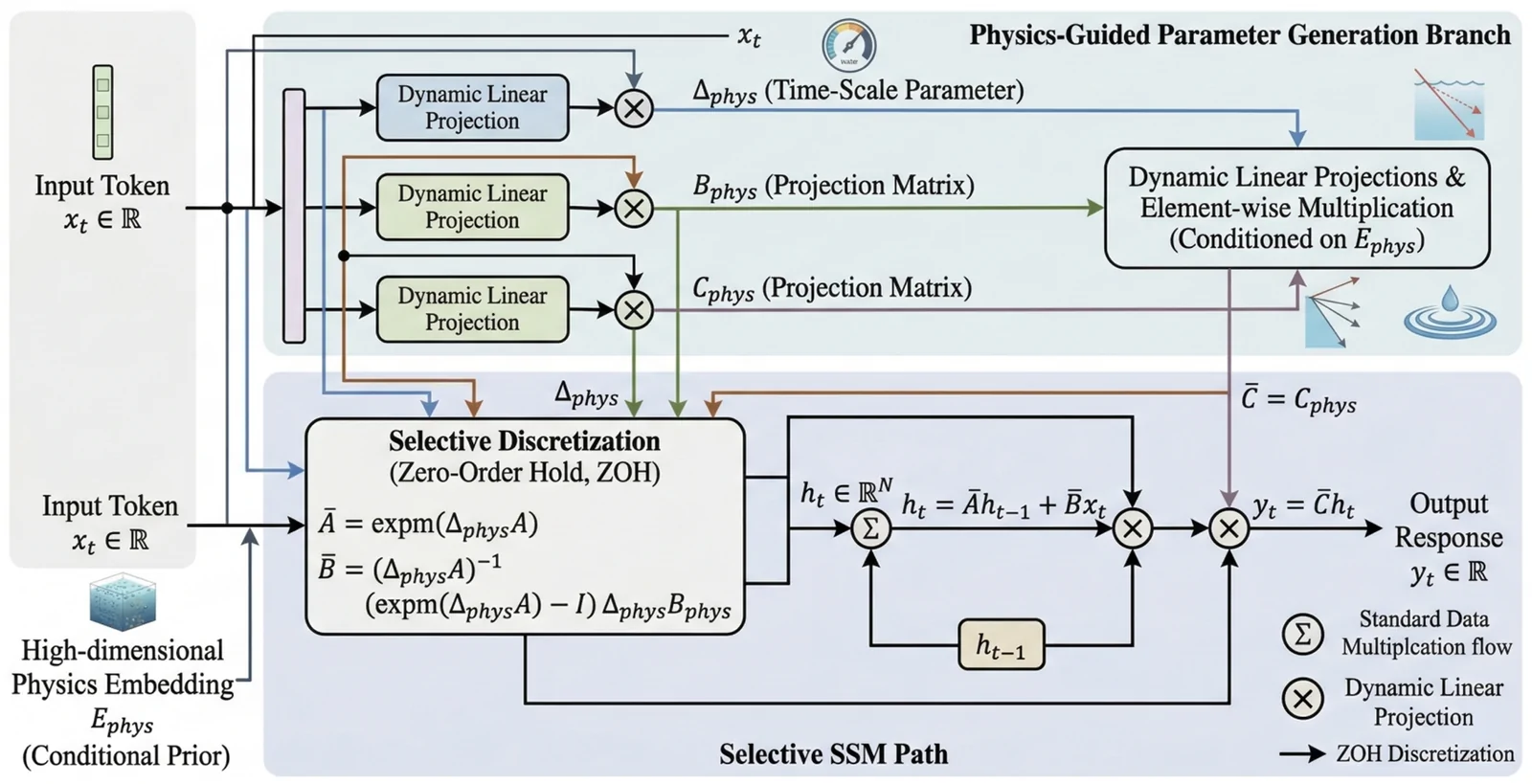
Detailed structure of the physics-guided selective state-space model.

A central component of Phys-Mamba is the Physics-Guided Selective State-Space Model, whose detailed structure is shown in Fig 3. In the standard Mamba formulation, the continuous state-space model is discretized using input-dependent parameters Δ, *B*, and *C*. We introduce dynamic reparameterization conditioned on the Physics Embedding:


$$\begin{array}{ccc} \Delta^{\prime } & = & \Delta ⊙\text{Linear}(E_{phys}), \end{array}$$ (3)

$$\begin{array}{ccc} B^{\prime } & = & B⊙\text{Linear}(E_{phys}), \end{array}$$ (4)

$$\begin{array}{ccc} C^{\prime } & = & C⊙\text{Linear}(E_{phys}) \end{array}$$ (5)

where Linear(·) denotes a lightweight linear projection followed by element-wise multiplication. Eqs. (3)–(5) describe the conceptual conditioning form. For implementation stability, the physics embedding is first normalized by LayerNorm and then projected to the same channel dimension as the corresponding selective-scan parameters. For a feature sequence of length *L* and channel dimension *d*, the projected embedding is broadcast or reshaped to match the parameter tensor before element-wise modulation. In practice, the modulation is implemented as a bounded residual gate rather than an unconstrained scaling:


$$\begin{array}{ccc} g_{\Delta } & = & 1+\alpha \tanh(W_{\Delta }E_{phys}),\\ g_{B} & = & 1+\alpha \tanh(W_{B}E_{phys}),\\ g_{C} & = & 1+\alpha \tanh(W_{C}E_{phys}), \end{array}$$

where α is a small scalar hyperparameter controlling the maximum modulation amplitude. In our implementation, α was set to 0.2. The modulated parameters are then computed as $\Delta^{\prime }=\Delta ⊙g_{\Delta }$, $B^{\prime }=B⊙g_{B}$, and $C^{\prime }=C⊙g_{C}$. The discretization follows the standard Mamba implementation, in which Δ remains positive through the original softplus parameterization. Therefore, the physics branch perturbs the selective-scan dynamics within a bounded range instead of changing the stability structure of the state-space model.

Each Physics-Guided Mamba Block follows the standard selective SSM architecture [21] (LayerNorm + linear projection + SiLU activation + selective scan), with the physics-modulated parameters $\Delta^{\prime }$, $B^{\prime }$, and $C^{\prime }$ used in the selective scan step. This design makes the selective scan conditioned on scene-specific degradation cues while retaining the linear *O*(*N*) computational complexity. This modulation is expected to make the selective scan more sensitive to degradation-related cues, although direct visualization of the internal allocation behavior remains a topic for future analysis. Unlike recent Mamba variants [22–27] that mainly introduce degradation information through auxiliary branches, guidance modules, spectral/wavelet processing, or loss-level constraints, our approach implements an explicit parameter-level integration and helps suppress color casts, over-enhancement, and non-physical artifacts.

### Dynamic cross-scale fusion module (CSFM)

**Fig 4:**
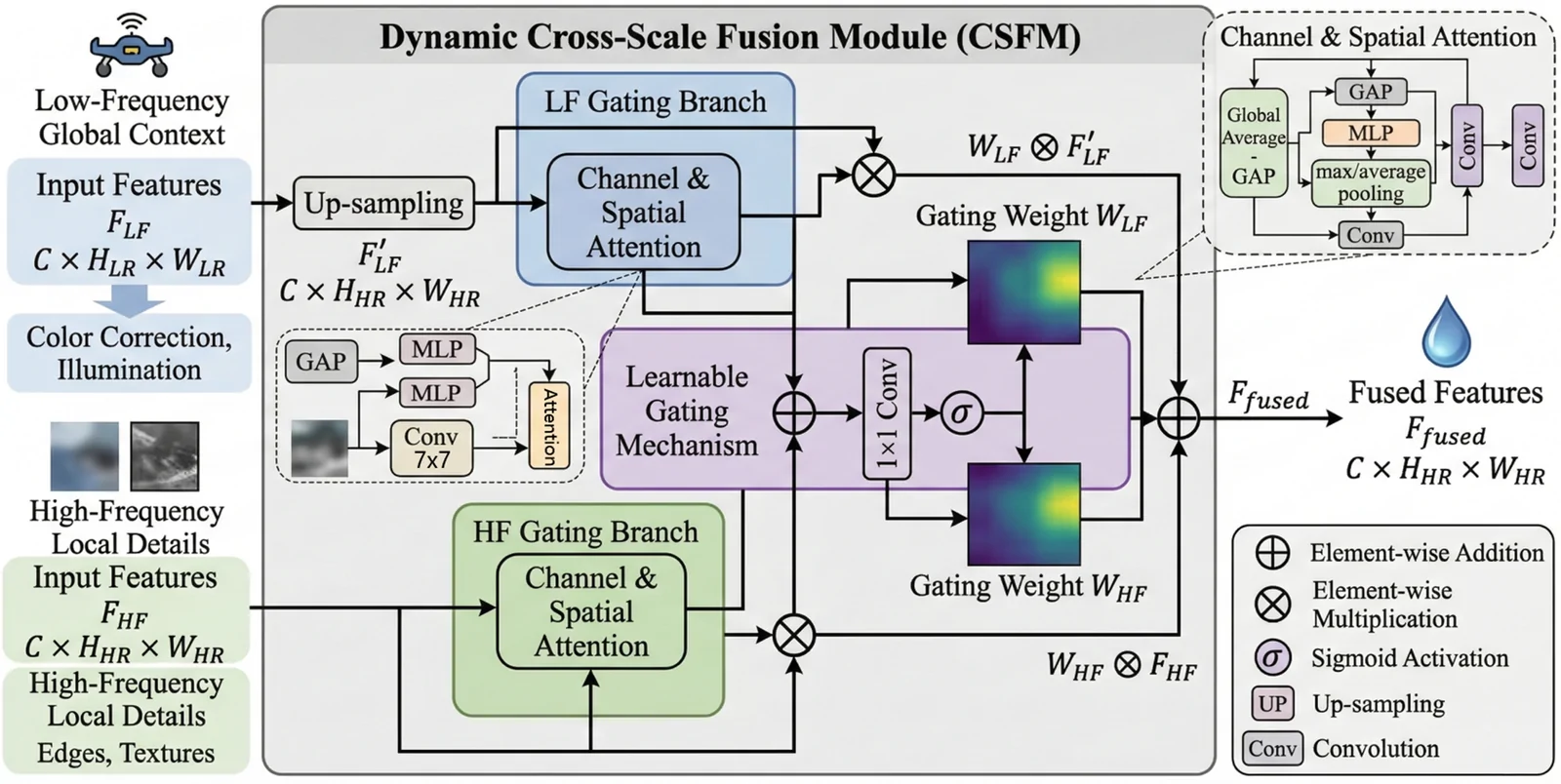
Schematic diagram of the dynamic cross-scale fusion module (CSFM).

Underwater degradation exhibits pronounced scale heterogeneity: low-resolution features primarily contain global color and illumination bias, while high-resolution features preserve local scattering-induced texture blur. Traditional skip-connection concatenation leads to exponential channel growth and severe parameter redundancy. To address this, we propose the Dynamic Cross-Scale Fusion Module (CSFM), whose schematic is shown in Fig 4.

CSFM employs a dual-branch attention-based gating mechanism. For low-frequency (LF) and high-frequency (HF) feature maps $F_{LF}$ and $F_{HF}$, the gating weights are independently generated as:


$$\begin{array}{c} W_{LF}=\sigma ({\text{Conv}}_{1\times 1}(\text{AvgPool}(F_{LF})⊕\text{MaxPool}(F_{LF}))) \end{array}$$ (6)

$$\begin{array}{c} W_{HF}=\sigma ({\text{Conv}}_{1\times 1}(\text{AvgPool}(F_{HF})⊕\text{MaxPool}(F_{HF}))) \end{array}$$ (7)

The final fused feature is computed via voxel-wise weighted summation:


$$\begin{array}{c} F_{fusion}=W_{LF}⊙F_{LF}+W_{HF}⊙F_{HF} \end{array}$$ (8)

This adaptive gating strategy performs cross-scale aggregation without increasing channel dimensions, supporting fine-detail recovery up to 4K resolution while keeping the total parameter count below 10M.

### High-fidelity loss function

To simultaneously enforce physical consistency, perceptual quality, and sharp detail reconstruction, we design a composite high-fidelity loss:


$$\begin{array}{c} {ℒ}_{total}=\lambda_{1}{ℒ}_{phys}+\lambda_{2}{ℒ}_{LPIPS}+\lambda_{3}{ℒ}_{charbonnier} \end{array}$$ (9)

where $\lambda_{1}=1.0$, $\lambda_{2}=0.01$, $\lambda_{3}=0.1$ (determined via grid search on the validation set), and the Charbonnier smooth-*L*_1_ loss uses $\epsilon =1\times 10^{-6}$. The physical reconstruction term ${ℒ}_{phys}$ penalizes inconsistency with the UIFM approximation, ${ℒ}_{LPIPS}$ aligns high-level semantics with human perception, and ${ℒ}_{charbonnier}$ preserves sharp edges and textures. This joint formulation encourages physically consistent high-fidelity reconstruction without assuming exact recovery of optical parameters.

## Experiments

### Experimental setup

All models are implemented in PyTorch 2.0 using the AdamW optimizer with an initial learning rate of $1\times 10^{-4}$ and cosine annealing decay. Training employs a batch size of 16 for 200 epochs with early stopping (patience of 10 epochs on validation PSNR). Datasets are split as follows: EUVP 7:1:2, UCHN/LSUI/Synthetic Deep-Sea 8:1:1. Input images are normalized to [0,1] with random crop, horizontal flip, and brightness jitter augmentation. All efficiency tests are conducted on an NVIDIA RTX 4090 (24 GB VRAM) in FP16 precision, batch size 1, with pre- and post-processing included. We compare Phys-Mamba against classical baselines (FUnIE-GAN [1], Ucolor [3], U-shape Transformer [16]) as well as six recent Mamba-based methods (UWMamba [22], PixMamba [23], UIEMamba [24], Mamba-enhanced Wavelet [25], FACMamba [26], and Mamba-Conv Hybrid [27]).

For reproducibility, fixed training/validation/test splits were used for all datasets. EUVP was divided into 8,400 training images, 1,200 validation images, and 2,400 test images following the 7:1:2 ratio. UCHN was divided into 800 training images, 100 validation images, and 100 test images. LSUI was divided into 4,000 training images, 500 validation images, and 500 test images. Synthetic Deep-Sea was divided into 2,400 training images, 300 validation images, and 300 test images. For paired datasets, the input and reference images were split at the pair level to avoid leakage. For unpaired data, image-level splitting was performed before training batch construction so that no image appeared in more than one split. No test images were used during training, validation, or hyperparameter selection. The split files and evaluation scripts will be released with the code to facilitate reproducibility.

For fair comparison, all retrainable baselines were trained using the same training/validation/test splits, input resolution, data augmentation strategy, optimizer setting, and evaluation scripts as Phys-Mamba unless otherwise specified. FUnIE-GAN [1], Ucolor [3], U-shape Transformer [16], UWMamba [22], PixMamba [23], UIEMamba [24], Mamba-enhanced Wavelet [25], FACMamba [26], and Mamba-Conv Hybrid [27] were evaluated under the same preprocessing and metric computation protocol. When official pretrained checkpoints were available, we used the released checkpoints and re-evaluated them on our fixed test splits; otherwise, the models were retrained using the official implementations with the same augmentation and training schedule. This setting was adopted to reduce the influence of split differences and implementation-specific metric computation.

**Fig 5:**
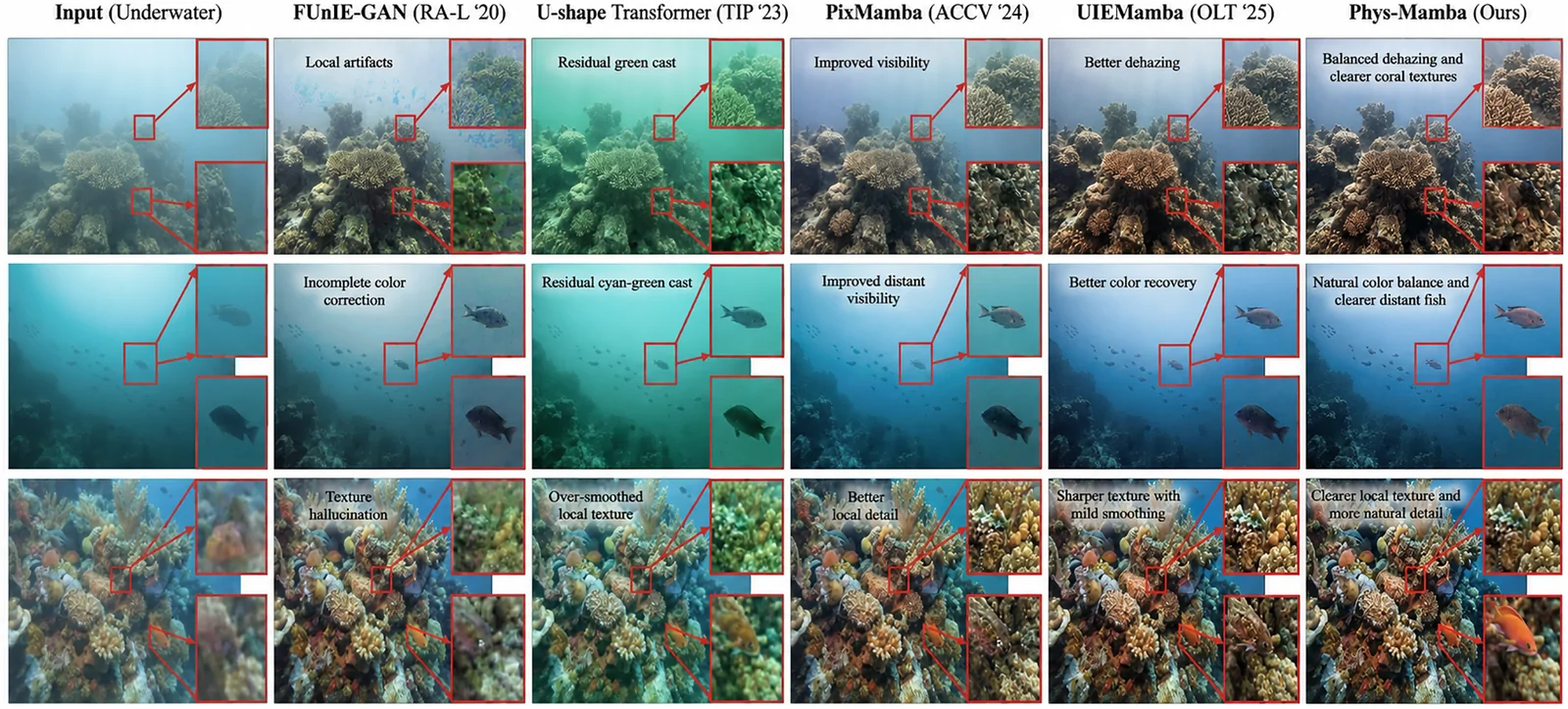
Qualitative comparison on representative real-world underwater images. From left to right, the columns show the degraded input, FUnIE-GAN, U-shape Transformer, PixMamba, UIEMamba, and Phys-Mamba. Red boxes and enlarged regions use identical spatial locations for all methods. FUnIE-GAN shows local artifacts or incomplete color correction in the displayed cases; U-shape Transformer retains residual green/cyan color casts and some over-smoothing; PixMamba and UIEMamba provide progressively improved visibility, dehazing, and detail recovery; and Phys-Mamba achieves a more balanced combination of haze removal, natural color, and local-texture preservation. No method-specific manual post-processing was applied.

**Fig 6:**
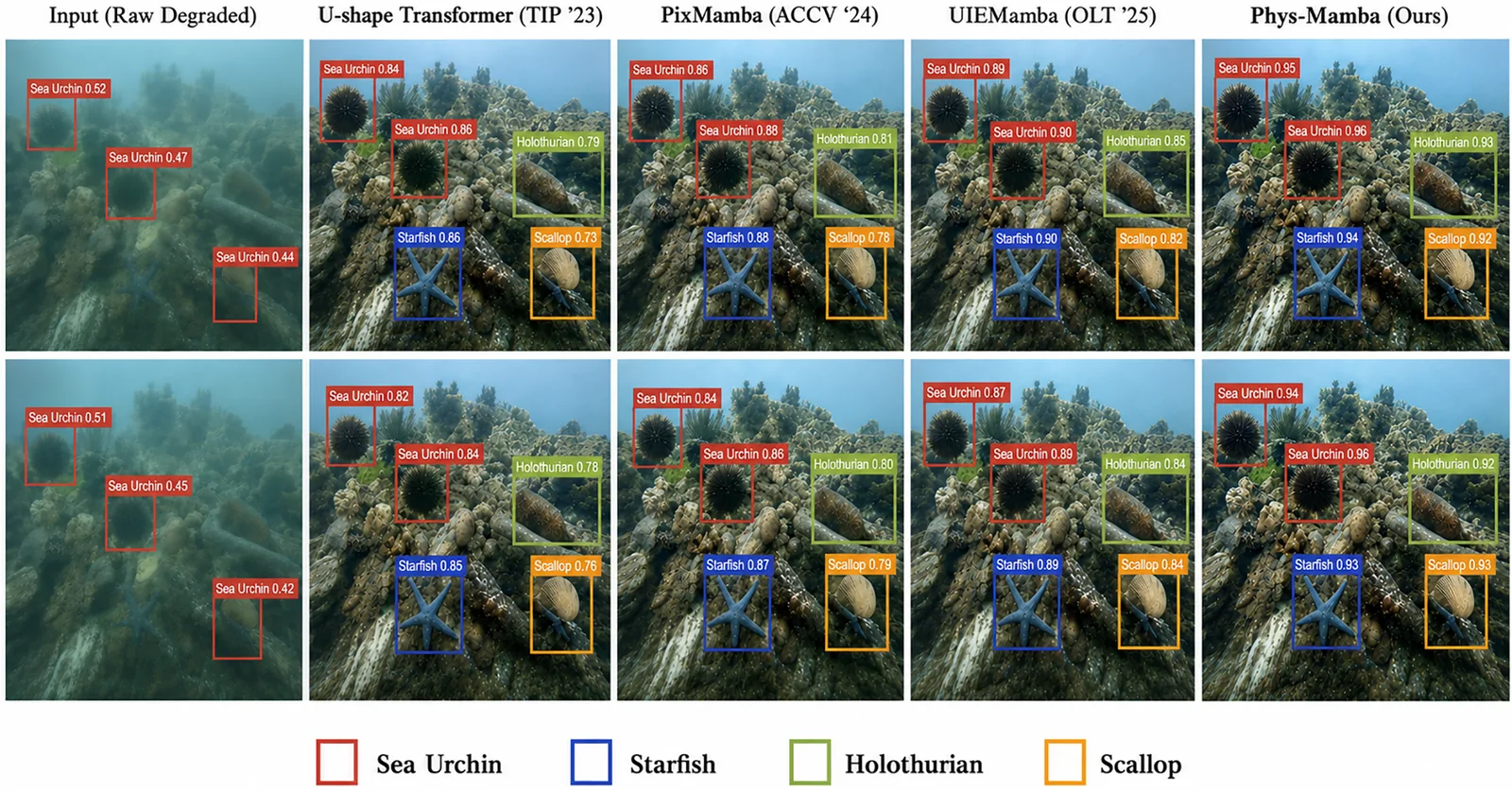
Representative YOLOv8 detection results on raw degraded images and images restored by U-shape Transformer, PixMamba, UIEMamba, and Phys-Mamba. The same pretrained detector, input size, confidence threshold (0.25), non-maximum-suppression settings, class definitions, and annotations were used for all panels. The colored boxes denote sea urchin, starfish, holothurian, and scallop detections, and the displayed confidence labels are per-instance outputs for these two examples. Phys-Mamba yields the highest confidence range and the clearest recovery of the four object categories in the displayed cases; the overall downstream conclusion is based on the full-test-set mAP results in Table 3.

For the qualitative comparisons in Figs 5 and 6, the representative test images were selected before side-by-side visual inspection and were processed using the same model checkpoints and inference settings as those used for the quantitative evaluation. The same input image, spatial resolution, and crop coordinates were used for every method, and no manual color adjustment, sharpening, denoising, or other post-processing was applied to individual outputs. The enlarged regions in Fig 5 therefore correspond to identical image locations across methods. For Fig 6, all restored images were passed to the same pretrained YOLOv8 detector using the same input size, confidence threshold of 0.25, non-maximum-suppression settings, class labels, and UDD annotations. The confidence values shown in the figure are per-instance outputs for the displayed examples; the mAP values in Table 3 were computed over the complete fixed test set.

### Quantitative evaluation

Table 1 presents the comprehensive quantitative results across multiple datasets and metrics. Phys-Mamba achieves competitive performance across both full-reference and non-reference metrics while maintaining a compact parameter count. Notably, it improves PSNR and SSIM compared with the evaluated recent Mamba-based competitors on the fixed test splits. Although UIQM and UCIQE are widely used non-reference metrics for underwater image enhancement, they may favor contrast or saturation changes that do not always correspond to physical fidelity. Therefore, we interpret these metrics together with PSNR, SSIM, downstream detection results, qualitative comparisons, and the limitations discussed below.

**Table 1:** Comprehensive quantitative comparison across multiple datasets and metrics. Best results are shown in bold.

Model	EUVP			UCHN			Synthetic Deep-Sea			LSUI		
	PSNR	SSIM	UIQM	PSNR	SSIM	UIQM	PSNR	SSIM	UIQM	PSNR	SSIM	UIQM
FUnIE-GAN [1]	24.52	0.816	2.92	23.10	0.792	2.85	22.45	0.780	2.78	23.80	0.805	2.90
Ucolor [3]	25.48	0.842	3.01	24.65	0.818	2.94	23.90	0.805	2.87	24.70	0.820	2.98
U-shape Transformer [16]	26.85	0.887	3.24	25.92	0.863	3.15	25.10	0.850	3.08	26.20	0.868	3.19
UWMamba [22]	26.14	0.841	3.02	25.40	0.825	2.98	24.80	0.810	2.95	25.60	0.835	3.00
PixMamba [23]	26.59	0.872	3.11	25.85	0.855	3.05	25.20	0.840	3.02	26.00	0.860	3.08
UIEMamba [24]	27.05	0.885	3.18	26.30	0.868	3.12	25.70	0.855	3.10	26.50	0.872	3.15
Mamba-enhanced Wavelet [25]	26.78	0.868	3.09	26.10	0.850	3.04	25.40	0.845	3.01	26.10	0.858	3.07
FACMamba [26]	26.92	0.879	3.15	26.15	0.862	3.08	25.55	0.852	3.06	26.30	0.865	3.12
Mamba-Conv Hybrid [27]	27.01	0.882	3.17	26.25	0.865	3.10	25.65	0.857	3.09	26.40	0.870	3.14
**Phys-Mamba (Ours)**	**28.12**	**0.915**	**3.42**	**27.30**	**0.892**	**3.35**	**26.50**	**0.878**	**3.28**	**27.45**	**0.901**	**3.38**

### Ablation study

To verify the contribution of each key component, we conduct detailed ablation experiments on EUVP and UCHN datasets. The results are summarized in Table 2. All variants share the same training settings as the full model.

**Table 2:** Ablation study on key components (EUVP and UCHN datasets). Best results in bold.

Model Variant	EUVP			UCHN		
	PSNR ↑	SSIM ↑	UIQM ↑	PSNR ↑	SSIM ↑	UIQM ↑
Baseline (Pure Mamba w/o physics or CSFM)	24.80	0.820	2.98	23.50	0.795	2.88
+ Physics Embedding Module (PEM) only	26.58	0.868	3.20	25.28	0.842	3.08
+ Dynamic CSFM only (no physics guidance)	26.05	0.852	3.12	24.85	0.830	3.02
+ Full Dynamic Modulation (Δ, *B*, *C* all modulated)	27.45	0.902	3.35	26.60	0.875	3.25
**Phys-Mamba (Ours, full)**	**28.12**	**0.915**	**3.42**	**27.30**	**0.892**	**3.35**

The ablation results show that the Physics Embedding Module (PEM) provides the largest single-component gain (+1.78 dB PSNR on EUVP) under the current setting, suggesting the usefulness of physics-informed guidance. CSFM further enhances multi-scale detail recovery (+0.47 dB incremental). Full dynamic modulation of all selective scan parameters (Δ, *B*, *C*) yields additional improvements. These results support the contribution of the main components under the current ablation setting. However, more fine-grained ablations are still needed to fully isolate the individual effects of Δ, *B*, and *C* modulation.

### Qualitative evaluation

Fig 5 presents representative qualitative comparisons on three real-world underwater scenes and, in response to the need for stronger visual evidence, includes two recent Mamba-based baselines, PixMamba [23] and UIEMamba [24], in addition to FUnIE-GAN [1] and U-shape Transformer [16]. All methods use the same input images and identical enlarged regions. In the first scene, FUnIE-GAN improves global contrast but introduces local artifacts, while U-shape Transformer retains a noticeable green cast. PixMamba recovers clearer scene structure, and UIEMamba provides stronger dehazing; Phys-Mamba produces the most balanced combination of haze removal, natural color, and coral-texture preservation among the displayed outputs. In the second scene, PixMamba and UIEMamba improve the visibility of the distant fish relative to the CNN/GAN and Transformer baselines, whereas Phys-Mamba further reduces the cyan-green bias and preserves a more natural color balance. In the third scene, FUnIE-GAN exhibits locally irregular textures and U-shape Transformer tends to smooth fine structures. PixMamba and UIEMamba recover progressively sharper local details, while Phys-Mamba retains clearer coral boundaries and more natural fine-scale appearance in the selected regions.

These observations are consistent with the quantitative ranking in Table 1, where Phys-Mamba obtains higher PSNR, SSIM, and UIQM values than the evaluated Mamba-based competitors on the fixed test splits. Nevertheless, Fig 5 contains representative examples rather than an exhaustive survey of all water types and degradation patterns, and it is therefore interpreted together with the full quantitative evaluation.

### Downstream tasks

For downstream evaluation, YOLOv8 was used to assess whether restoration-based preprocessing improves underwater object detection. The same pretrained YOLOv8 detector was directly applied to raw degraded images and to images restored by different preprocessing methods without retraining. The detector architecture, annotation labels, confidence threshold, non-maximum-suppression settings, and evaluation protocol were kept identical across all image sources. The confidence threshold was set to 0.25, and mAP@0.5 was computed using the same UDD test annotations for all methods. This controlled design isolates the influence of image restoration and prevents the reported improvement from being interpreted as a change to the detector itself.

As shown in Table 3, the raw degraded images yield an mAP@0.5 of 49.0%. U-shape Transformer, PixMamba, and UIEMamba increase this value to 62.1%, 62.6%, and 64.2%, respectively, whereas Phys-Mamba reaches 69.7%, corresponding to an improvement of 20.7 percentage points over the raw input. Fig 6 adds PixMamba and UIEMamba to the visual comparison and shows two representative test examples under the same detector settings. In these displayed examples, the per-instance confidence values span 0.42–0.52 for the detections retained on raw images, 0.73–0.86 after U-shape Transformer preprocessing, 0.78–0.88 after PixMamba preprocessing, 0.82–0.90 after UIEMamba preprocessing, and 0.92–0.96 after Phys-Mamba preprocessing. The restored images also enable the fixed detector to recover starfish, holothurian, and scallop instances that are not retained above threshold in the corresponding raw-image panels. These example-level confidence values are illustrative and should be interpreted together with the full-test-set mAP results in Table 3.

**Table 3:** Downstream object detection performance (mAP@0.5) on the UDD dataset.

Image Source	Sea Urchin	Holothurian	Scallop	Starfish	mAP@0.5 (%) ↑
Raw Degraded	48.2	42.1	50.5	55.3	49.0
FUnIE-GAN [1]	55.4	48.6	58.2	61.4	55.9
U-shape Transformer [16]	60.1	55.3	64.8	68.2	62.1
UWMamba [22]	58.7	52.4	62.1	66.5	60.4
PixMamba [23]	61.2	54.8	65.3	69.1	62.6
UIEMamba [24]	62.5	56.9	66.8	70.4	64.2
**Phys-Mamba (Ours)**	**68.5**	**63.4**	**71.2**	**75.6**	**69.7**

### Efficiency and real-time analysis

Table 4 reports computational efficiency on an NVIDIA RTX 4090. Phys-Mamba achieves 34 FPS at 4K resolution with 2.8 GB GPU memory under FP16 and batch size 1. These results demonstrate efficient high-resolution inference on a high-end desktop GPU. However, because RTX 4090 is not an embedded AUV/ROV platform, the reported efficiency should not be interpreted as an embedded-hardware result. Evaluation on Jetson-class or other power-constrained hardware remains future work.

**Table 4:** Efficiency comparison (RTX 4090, FP16, batch size 1, including pre/post-processing).

Method	Params (M)	FLOPs@4K (G)	FPS@1080p	FPS@4K	GPU Mem@4K (GB)
U-shape Transformer [16]	45.3	OOM	12	N/A	OOM
UWMamba [22]	7.8	185	78	22	3.9
PixMamba [23]	8.7	172	82	25	3.5
UIEMamba [24]	8.2	178	80	24	3.6
Mamba-Conv Hybrid [27]	8.9	169	85	27	3.2
**Phys-Mamba (Ours)**	**8.6**	**168**	**95**	**34**	**2.8**

### Robustness under extreme conditions

To examine generalization, we additionally evaluated the model on difficult subsets defined by low UCIQE values, low brightness, and high-resolution inputs. Phys-Mamba showed a smaller performance decrease than the compared methods in this setting. Nevertheless, these subsets do not fully cover all challenging underwater conditions, such as strong artificial lighting, dense suspended particles, close-range backscatter, sand/mud turbidity, night-time scenes, or scenes dominated by red objects. Therefore, the robustness claim is limited to the evaluated subsets.

Collectively, the quantitative, qualitative, ablation, downstream, efficiency, and robustness results suggest that Phys-Mamba partially alleviates the trade-off among physical fidelity, global modeling capability, and high-resolution inference efficiency.

## Discussion

### Performance gains and mechanistic insights

The experimental results presented in this study indicate that Phys-Mamba can partially alleviate the trade-off among physical fidelity, global modeling capability, and computational efficiency in underwater image restoration. As shown in Table 1, our model achieves competitive performance across diverse benchmarks (EUVP, UCHN, Synthetic Deep-Sea, and LSUI), with improvements over the evaluated classical baselines and recent Mamba-based methods [22–27] on the fixed test splits.

These gains may be related to the explicit parameter-level coupling between UIFM-inspired latent degradation variables and Mamba’s selective scan mechanism (Δ, *B*, *C*). Instead of treating the estimated variables as calibrated optical measurements, Phys-Mamba uses them as scene-adaptive cues that encourage the sequence evolution to remain consistent with the simplified UIFM formulation. This modulation helps suppress color casts and over-enhancement while preserving long-range dependencies with linear *O*(*N*) complexity.

Qualitative results in Fig 5 provide a direct comparison with both conventional and recent Mamba-based methods. In the displayed scenes, FUnIE-GAN retains local artifacts or incomplete color correction, and U-shape Transformer exhibits residual green/cyan casts or over-smoothed local structures. PixMamba and UIEMamba substantially improve visibility and texture recovery, confirming that selective state-space modeling is effective for underwater restoration. Relative to these Mamba baselines, Phys-Mamba shows a more balanced combination of dehazing, color correction, and preservation of coral and fish details. This visual trend is consistent with the quantitative improvements reported in Table 1, although the examples do not establish superiority under every possible underwater condition.

### Component contributions and ablation insights

The ablation study (Table 2) examines the contributions of the main components across EUVP and UCHN datasets. Introducing the Physics Embedding Module (PEM) alone yields a + 1.78 dB PSNR gain on EUVP (from 24.80 dB baseline to 26.58 dB), suggesting that UIFM-inspired degradation cues are useful in this setting. The Dynamic Cross-Scale Fusion Module (CSFM) alone provides a + 1.25 dB improvement on EUVP by addressing the scale-heterogeneous nature of underwater degradation (global illumination bias at low resolution versus local scattering blur at high resolution). Adding full dynamic modulation of all selective scan parameters (Δ, *B*, and *C*) further improves performance to 27.45 dB. Their full combination achieves 28.12 dB PSNR and 0.915 SSIM on EUVP. These results support the contribution of the main components under the current ablation setting. However, more fine-grained ablations are still needed to fully isolate the individual effects of Δ, *B*, and *C* modulation.

This design distinguishes Phys-Mamba from recent Mamba-UIE works [24–27] by placing degradation cues at the selective-scan parameter level. The current results support this design choice, while additional variants will be needed to fully separate the effects of the individual parameter modulations.

### Practical implications for underwater robotics

Beyond image quality, Phys-Mamba shows potential as a preprocessing module for underwater robotic perception. Under the controlled protocol in Table 3, it improves YOLOv8 mAP@0.5 by 20.7 percentage points relative to raw degraded images and exceeds the evaluated U-shape Transformer, PixMamba, and UIEMamba preprocessing results by 7.6, 7.1, and 5.5 percentage points, respectively. The representative examples in Fig 6 show the same ordering in per-instance confidence and illustrate improved recovery of sea urchins, starfish, holothurians, and scallops after Phys-Mamba restoration. These results suggest that improved color balance and edge visibility can benefit a fixed detector, particularly for small or visually obscured underwater objects. However, the downstream findings remain specific to the adopted detector, UDD test set, and no-retraining preprocessing protocol. Efficiency metrics (Table 4) further show that the model supports 34 FPS 4K inference with 2.8 GB GPU memory on a high-end desktop GPU. Because this hardware is not an embedded AUV/ROV platform, deployment on power-constrained onboard hardware remains future work.

### Future research directions

Future research will focus on improving temporal consistency, expanding physical modeling beyond the simplified UIFM, and validating deployment-oriented inference on realistic underwater robotic hardware.

Specific directions include: (1) extending the method to spatiotemporal Video-Mamba by incorporating optical flow-guided temporal consistency and inter-frame degradation-cue propagation; (2) developing a hybrid nonlinear scattering model fused with Retinex-based low-light enhancement to improve robustness in more challenging deep-sea conditions; (3) integrating sonar depth maps and water turbidity sensors for adaptive prior calibration; and (4) exploring unsupervised domain-adaptive training using unlabeled real-world AUV footage to improve generalization across previously unseen water bodies.

### Limitations

This study has several limitations. First, the estimated transmission map and ambient light are used as UIFM-inspired latent degradation variables rather than calibrated physical measurements, because ground-truth optical parameters are not available in the adopted datasets. Second, the classical UIFM assumes a simplified linear scattering process and may not fully describe scenes with strong artificial lighting, dense suspended particles, severe backscatter, night-time imaging, or highly non-uniform illumination. Therefore, the proposed method is mainly intended for medium-to-low turbidity natural underwater scenes, and its behavior under more extreme imaging conditions requires further validation. Third, although Phys-Mamba achieves efficient 4K inference on an RTX 4090, embedded deployment on realistic AUV/ROV hardware such as Jetson-class devices remains future work. Finally, the current ablation study verifies the main components, but more fine-grained variants, such as separately modulating Δ, *B*, and *C*, using the physical loss without scan modulation, replacing the physics branch with simple concatenation, replacing CSFM with standard skip connections, or removing LPIPS, will be investigated in future work.

## Conclusion

In conclusion, Phys-Mamba provides a physics-informed Mamba-based framework for underwater image restoration by coupling UIFM-inspired latent degradation variables with selective-scan parameter modulation and dynamic cross-scale fusion. Experimental results suggest that this design can improve restoration quality and maintain efficient high-resolution inference on a high-end GPU. At the same time, the method relies on a simplified UIFM assumption, and further validation on embedded hardware, more diverse underwater conditions, and fine-grained ablation settings remains necessary. We believe this work offers a useful step toward physically guided and efficient underwater visual restoration.

## Supporting information

S1 File Supplementary experimental materials. The ZIP archive contains supporting files associated with the experiments reported in this study.
(ZIP)

S2 File Data final submission. (ZIP)
